# The Effect of *FLT1* Variant on Long-Term Cardiovascular Outcomes: Validation of a Locus Identified in a Previous Genome-Wide Association Study

**DOI:** 10.1371/journal.pone.0164705

**Published:** 2016-10-13

**Authors:** Chan Joo Lee, Ji-Young Lee, Chi-Yoon Oum, Jong-Chan Youn, Seok-Min Kang, Donghoon Choi, Yangsoo Jang, Sungha Park, Sun Ha Jee, Sang-Hak Lee

**Affiliations:** 1 Division of Cardiology, Department of Internal Medicine, Yonsei University College of Medicine, Seoul, Korea; 2 Cardiovascular Research Institute and Cardiovascular Genome Center, Yonsei College of Medicine, Seoul, Korea; 3 Department of Biostatistics and Computing, the Graduate School, Yonsei University, Seoul, Korea; 4 Institute of Health Promotion, Graduate School of Public Health, Yonsei University, Seoul, Korea; National Cancer Institute, UNITED STATES

## Abstract

**Background:**

Data on genetic variants that can predict follow-up cardiovascular events are highly limited, particularly for Asians. The aim of this study was to validate the effects of two variants in *FLT1* and 9p21 on long-term cardiovascular outcomes in high-risk Korean patients.

**Methods:**

We examined the prognostic values of the rs9508025 and rs1333049 variants that were found to be associated with coronary artery disease (CAD) risk in a previous Korean genome-wide association study. A total of 2693 patients (mean age: 55.2 years; male: 55.2%) with CAD or its risk factors at baseline were enrolled and followed for major adverse cardiac events (MACE).

**Results:**

During the mean follow-up of 8.8 years, 15.4% of the patients experienced MACE. Kaplan-Meier curves showed that MACE-free survival was different according to the genotype of rs9508025 (log rank p = 0.02), whereas rs1333049 genotype did not correlate with the prognosis. Multivariate Cox proportional hazard analysis showed that C-allele of rs9508025 was significantly associated with a high rate of MACE, while rs1333049 was not. Further analyses demonstrated that the association of the rs9508025 variant with MACE was mainly due to its relation to coronary revascularization, which was also associated with the rs1333049 variant. In an additional analysis, rs9508025 was found to be an independent determinant of the outcome only in the subgroup with history of CAD.

**Conclusions:**

rs9508025 in *FLT1* was significantly associated with long-term cardiovascular events, particularly in patients with prior CAD. The association of rs1333049 in 9p21 was not significant.

## Introduction

In order to predict the risk for atherosclerotic cardiovascular events, one of the most common causes of mortality and morbidity around the world, numerous studies have been performed. An accurate risk assessment using effective markers can be helpful for preventing cardiovascular events [1]. For instance, adding the influence of single nucleotide polymorphisms (SNPs) to the conventional risk calculation has been used for improving risk discrimination [2, 3].

Atherosclerotic cardiovascular disease is known to be under strong genetic influence. For decades, significant relationship between several SNPs in candidate genes and cardiovascular risk was reported by association studies [4]. In addition, with the progression of high-throughput technology and cost reductions, large-scale genome-wide association studies (GWAS) revealed multiple disease-related loci and pathways, provided researchers with new pathophysiological insights, and risk markers as well [5]. Genetic variants linked to traditional risk factors are found among dozens of validated risk variants. However, many of them, such as those in 9p21, are not related to the known risk factors [6].

To date, most genetic marker investigation have been based on association studies in a cross-sectional manner. Conversely, several studies that searched for the genetic determinants associated with the follow-up outcome were conducted. Among the individuals in need of primary cardiovascular prevention, a variant at 3q22.3 [7] and another one in eNOS [8] were identified as having predictive value in longitudinal studies. In patients that already experienced cardiovascular events, genetic variants in lipoprotein-associated genes [7, 9–11], 9p21 [12], and ABO blood group system [13] were found to have prognostic values. However, data regarding variants validated for predicting long-term cardiovascular outcome are still extremely limited. In Asians, a few association studies have identified several variants related to the risk of coronary artery disease [14, 15]. However, studies on the variants predicting cardiovascular risk in individuals of Asian ethnicities had several limitations: 1) they have been far less studied than the individuals of European ancestry, 2) among the studies on Asians, the investigated variants that were predictive of follow-up outcomes were very rare, and 3) the follow-up duration for Asian studies performed thus far has not been long enough for valid conclusions regarding the association with the variants [16, 17].

The aims of this study were to validate the association of two variants on the long-term cardiovascular outcome in high-risk Korean patients, with or without coronary artery disease (CAD). To identify variants predictive of long-term cardiovascular outcomes, two candidate SNPs were selected. One of the candidates was rs9508025 in *FLT1*, because its p value was more significant than that of *SORT1* or *PDGFD* in Korean GWAS [18], although it was not very strong. Furthermore, it was replicated in the Japanese population, another East Asian ethnicity [18]. The loci of *SORT1* and *PDGFD* were reported to be associated with CAD in an international study and replicated in Koreans. However, the p values were between 7.9 x 10–4 and 1.3 x 10–3 when they were analyzed in Korean GWAS alone. That was why we did not include these two SNPs in the current study. In addition, we excluded loci in BRAP, because three SNPs at or near *BRAP* were at 12q24, which was reported to be a pleiotropic region in recent studies [19, 20]. Such regions may affect multiple phenotypes and make it difficult to clarify the independent effects in our study.

The association between the variants and the composite and individual component of major adverse cardiac events (MACE), during a mean follow-up of 8.8 years, were analyzed.

## Methods

### Study population

Between November 2000, and March 2011, 2693 study subjects were enrolled from Cardiovascular Genome Center, Yonsei University College of Medicine, Seoul, Korea. Men and women with either history of, or more than two risk factors for CAD were recruited. Risk factors included old age (men ≥45 years; women ≥55 years), history of hypertension (blood pressure 140/90 mmHg), diabetes mellitus (fasting blood glucose ≥126 mg/dL or hemoglobinA1c ≥6.5%), hyperlipidemia (low-density lipoprotein-cholesterol ≥130 mg/dL), and current smoker. Trained nurses obtained the clinical data, including demographic parameters and medical history. Patients underwent coronary angiography depending on the relevant chest symptoms. CAD was defined as a significant stenosis (≥50%) in at least one epicardial coronary artery. The Institutional Review Board of Severance Hospital, Yonsei University College of Medicine approved all study designs and protocols, and all participants provided their written informed consent.

### Genotyping

Genomic DNA was extracted from the peripheral blood samples by QuickGene SNP Kit DNA (Fuji film, Tokyo, Japan). Taqman genotyping was performed using ABI Prism 7000 (Applied Biosystems, Foster City, CA, USA). Blind duplicates (10%) revealed a 99.6% genotyping concordance rate, implying that a false discovery due to typing error was unlikely. The genotype frequencies of two SNPs were all in agreement with the Hardy-Weinberg equilibrium test (p >0.05).

### Outcome variables

The outcome variables were MACE, which include cardiovascular death, non-fatal myocardial infarction, coronary revascularization (percutaneous coronary intervention and coronary artery bypass graft), and stroke. Death was classified as cardiovascular if it was related to myocardial infarction or ischemia, arrhythmia, heart failure, or stroke. To assess MACE that occurred between patients’ enrollment and December 31, 2012, computerized searches for the cause of death were conducted using Korean National Health Insurance Corporation data. Other MACE were ascertained from bills with discharge diagnosis, of which nearly all are submitted to the Corporation. Prior Korean studies used this data to confirm long-term clinical outcome [5, 21]. When an individual experienced multiple MACE during the entire follow-up period, the first one was regarded as his or her MACE. The duration between the enrollment and the first MACE was used in the survival analyses.

### Statistical analysis

Continuous variables are presented as mean ± standard deviation and categorical variables are presented as frequencies and percentages. Baseline characteristics of the study subjects according to genotypes were compared by using analysis of variance for quantitative traits and the chi-square test for categorical variables. Triglyceride levels that have a skewed distribution were analyzed by Kruskal-Wallis test. Cumulative MACE-free survival curves for each genotype established by the Kaplan-Meier method were compared by the log-rank test. The Cox proportional hazard regression analysis was used to identify independent predictors for MACE and its individual components. Age, sex, and all available risk factors were adjusted for in the multivariate analysis. When variables not significant in univariate analysis are excluded in multivariate analysis, the model can be more informative. However, we included those variables by clinical reasoning, because they are known risk factors or confounders. In addition, we could not rule out the possibility that they might be associated with biological pathways. Therefore, we included the variables in multivariate analysis to control them. Hazard ratios and 95% confidence intervals were reported. Adjustment for multiple testing was performed by using Bonferroni correction. Our Bonferroni threshold was the α value divided by the number of independent tests (0.05/2 = 0.025). The determinants of MACE were further analyzed in the subgroups according to the history of CAD. All analyses used two-tailed tests with a significance level of 0.05. All statistical analyses were performed using SAS 9.4 (SAS institute, Cary, NC, USA).

## Results

### Characteristics of the study population

Out of 2693 patients (55.2 ± 11.0 years, male: 55.2%) enrolled in this study, 943 (35.0%) had history of CAD. Most of the study subjects (98.3%) were hypertensive. Clinical characteristics of the subjects classified based on the genotype of each SNP are displayed in Table 1. The frequency of hyperlipidemia was different between the patients with each genotype of rs9508025 (p = 0.045), while the prevalence of CAD differ between the patients with each genotype of rs1333049 (p<0.001).

**Table 1:** Baseline characteristics and clinical outcomes of the study population classified based on rs9508025 and rs1333049 genotype.

	Total (N = 2693)	rs9508025 (N = 2693)				rs1333049 (N = 2675)			
Variables		GG (N = 743)	CG (N = 1316)	CC (N = 634)	P value	GG (N = 718)	CG (N = 1310)	CC (N = 647)	P value
Age, years	55.2±11.0	54.8±11.1	55.4±10.9	55.3±11.3	0.48	55.4±11.3	55.2±11.0	55.1±10.8	0.87
Male	1486 (55.2)	418 (56.3)	717 (54.5)	351 (55.4)	0.74	337 (46.9)	580 (44.3)	280 (43.3)	0.35
Hypertension	2647 (98.3)	731 (98.4)	1291 (98.1)	625 (98.6)	0.73	706 (98.3)	1288 (98.3)	635 (98.2)	0.96
Diabetes mellitus	399 (14.8)	98 (13.2)	213 (16.2)	88 (13.9)	0.14	111 (15.5)	199 (15.2)	89 (13.8)	0.63
Hyperlipidemia	1111 (41.3)	309 (41.6)	516 (39.2)	286 (45.1)	0.045	297 (41.4)	544 (41.5)	263 (40.7)	0.93
Current smoker	391 (15.6)	102 (14.7)	186 (15.1)	103 (17.8)	0.27	106 (15.9)	197 (16.1)	86 (14.5)	0.66
CAD	943 (35.0)	249 (33.5)	472 (35.9)	222 (35.0)	0.56	243 (33.8)	424 (32.4)	268 (41.4)	<0.001
BMI, kg/m^2^	25.0±3.0	25.1±3.1	24.9±3.0	24.9±2.9	0.66	25.0±2.8	25.0±3.1	24.8±3.0	0.57
Total cholesterol, mg/dL	197±43	197±46	196±41	197±42	0.72	196±45	197±40	196±44	0.94
Triglyceride, mg/dL	135 (101)	135 (96)	133 (100)	138 (109)	0.58	130 (99)	137 (101)	134 (99)	0.13
HDL-cholesterol, mg/dL	43.9±11.5	43.7±11.1	44.0±12.1	44.0±11.3	0.82	44.3±11.6	43.7±11.7	43.9±11.7	0.61
MACE	416 (15.4)	96 (12.9)	202 (15.4)	118 (18.6)	0.01	112 (15.6)	187 (14.3)	114 (17.6)	0.16
Cardiovascular death	21 (0.8)	5 (0.8)	8 (0.6)	7 (1.0)	0.50	6 (0.8)	11 (0.8)	4 (0.6)	0.86
Nonfatal MI	119 (4.4)	29 (3.9)	59 (4.5)	31 (4.9)	0.67	30 (4.2)	52 (4.0)	36 (5.6)	0.25
Coronary revascularization	156 (5.8)	29 (3.9)	84 (6.4)	43 (6.8)	0.03	33 (4.6)	72 (5.5)	50 (7.7)	0.04
Nonfatal stroke	120 (4.5)	32 (4.3)	51 (3.9)	37 (5.8)	0.14	43 (6.0)	52 (4.0)	24 (3.7)	0.06

Data are presented as mean ± SD, median (interquartile range), or n (%); CAD, coronary artery disease; BMI, body mass index; HDL: high-density lipoprotein; MACE, major adverse cardiovascular events; MI, myocardial infarction.

### Association between the SNPs and cardiovascular events

During the mean follow-up of 8.8 years, 416 patients (15.4%) experienced MACE: 21 cardiovascular death (0.8%), 119 non-fatal myocardial infarction (4.4%), 156 coronary revascularization (5.8%), and 120 stroke (4.5%). The incidence of MACE was different between individuals with each genotype of rs9508025 (12.9%, 15.4%, and 18.6% of patients with GG, GC, and CC genotype, respectively, p = 0.01). No significant difference in MACE was found between subjects with different rs1333049 genotypes (15.6%, 14.3%, and 17.6% of patients with GG, GC, and CC genotype, respectively, p = 0.16).

### Determinants of cardiovascular events

**Fig 1:**
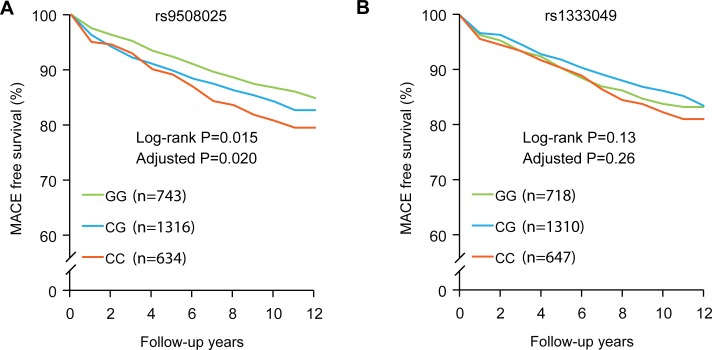
**Kaplan-Meier curves for long-term MACE-free survival of the study subjects depending on rs9508025 (A) and rs1333049 polymorphisms (B).** The proportion was significantly different among the individuals with different genotypes of rs9508025 (log rank p = 0.015, adjusted p = 0.020), but it was not significantly related to the genotype of rs1333049 (log rank p = 0.13, adjusted p = 0.26). Adjusted p values are based on multivariate Cox proportional hazards regression analysis that included variables of age, sex, hypertension, diabetes mellitus, hyperlipidemia, smoking status, CAD, and the SNPs.

Fig 1A and 1B shows the Kaplan-Meier curves for MACE-free survival. The proportion was significantly different among the individuals with different genotypes of rs9508025 (log rank p = 0.02), but it was not significantly related to the genotype of rs1333049 (log rank p = 0.13). Univariate and multivariate Cox proportional hazard analyses of the predictors of MACE are presented in Table 2. Age, male sex, diabetes mellitus, hyperlipidemia, prior CAD, and C allele of rs9508025 were associated with the higher MACE incidence. However, the genotype of rs1333049 did not correlate with the outcomes. After adjusting for age, sex, and all available risk factors, the rs9508025 variant was found to be a significantly associated with MACE (HR: 1.19, p = 0.02). Individual components of MACE were evaluated, and the results of multivariate Cox regression analysis are shown in Table 3. Age, sex, hypertension, diabetes, hyperlipidemia, smoking status, CAD, and the SNPs were included in the analysis. The association of the rs9508025 variant with MACE was mainly due to its relation to coronary revascularization (HR: 1.36, p = 0.02). Interestingly, the C allele of rs1333049 showed an association with the incidence of coronary revascularization as well (HR: 1.34, p = 0.02) (Table 3).

**Table 2:** Predictors of MACE determined by Cox regression analysis.

	Univariate model		Multivariate model	
Factors	HR (95% CI)	P	HR (95% CI)	P
Age	1.04 (1.03–1.05)	<0.001	1.03 (1.02–1.04)	<0.001
Male	1.53 (1.25–1.87)	<0.001	1.38 (1.09–1.73)	0.01
Hypertension	1.46 (0.61–3.52)	0.40	1.22 (0.50–3.00)	0.67
Diabetes Mellitus	2.08 (1.66–2.60)	<0.001	1.51 (1.17–1.94)	0.002
Hyperlipidemia	1.76 (1.45–2.14)	<0.001	1.19 (0.96–1.48)	0.11
Current smoker	1.10 (0.83–1.46)	0.50	1.07 (0.79–1.43)	0.66
CAD	2.92 (2.40–3.54)	<0.001	2.00 (1.60–2.50)	<0.001
rs9508025^a^	1.22 (1.07–1.39)	0.004	1.19 (1.03–1.38)	0.02
rs1333049^a^	1.07 (0.93–1.22)	0.34	1.09 (0.94–1.26)	0.26

MACE, major adverse cardiovascular events; HR, hazard ratio; CI, confidence interval; CAD, coronary artery disease.

^a^Additive genetic model.

**Table 3:** Predictors of the individual components of MACE in all patients (N = 2693).

	MACE		Cardiovascular death		Nonfatal MI		Coronary revascularization		Nonfatal stroke	
Factors	HR (95% CI)	P	HR (95% CI)	P	HR (95% CI)	P	HR (95% CI)	P	HR (95% CI)	P
Age	1.03 (1.02–1.04)	< .001	1.06 (1.01–1.12)	0.02	1.01 (0.99–1.03)	0.41	1.02 (0.99–1.04)	0.10	1.06 (1.04–1.08)	< .001
Male	1.38 (1.09–1.73)	0.01	3.61 (1.15–11.31)	0.002	2.13 (1.27–3.58)	0.004	1.38 (0.94–2.04)	0.10	0.91 (0.61–1.35)	0.64
Hypertension	1.22 (0.50–3.00)	0.67	NA	NA	0.70 (0.17–2.88)	0.62	2.31 (0.32–16.7)	0.41	1.08 (0.27–4.43)	0.91
Diabetes mellitus	1.51 (1.17–1.94)	0.002	0.75 (0.22–2.63)	0.66	2.11 (1.32–3.36)	0.002	1.25 (0.81–1.91)	0.32	1.54 (0.96–2.46)	0.07
Hyperlipidemia	1.19 (0.96–1.48)	0.11	5.72 (1.84–17.73)	0.003	0.95 (0.62–1.47)	0.83	1.33 (0.92–1.93)	0.13	1.01 (0.68–1.50)	0.96
Current smoker	1.07 (0.79–1.43)	0.66	0.87 (0.24–3.11)	0.51	1.65 (1.02–2.67)	0.04	0.72 (0.42–1.25)	0.24	1.00 (0.55–1.81)	0.99
CAD	2.00 (1.60–2.50)	< .001	1.21 (0.47–3.07)	0.69	2.43 (1.55–3.79)	< .001	2.00 (1.60–2.50)	< .001	0.75 (0.49–1.17)	0.21
rs9508025^a^	1.19 (1.03–1.38)	0.02	1.17 (0.62–2.18)	0.63	0.97 (0.73–1.31)	0.86	1.36 (1.06–1.75)	0.02	1.23 (0.95–1.60)	0.11
rs1333049^a^	1.09 (0.94–1.26)	0.26	0.93 (0.49–1.77)	0.82	1.21 (0.90–1.61)	0.21	1.34 (1.04–1.72)	0.02	0.81 (0.62–1.06)	0.12

MACE, major adverse cardiac events; MI, myocardial infarction; HR, hazard ratio; CI, confidence internal; CAD, coronary artery disease; NA, not available due to missing data.

^a^Additive genetic model.

CAD itself was associated with MACE and there seems to be an interaction among CAD, MACE, and rs9508025. We analyzed the determinants of MACE in the subgroups classified by the history of CAD, and found that the SNP was an independent determinant of MACE only in patients with CAD (S1 Fig, S1 Table). It was not significant in patients without CAD (S1 Table). rs1333049 did not show a significant association in both subgroups (S1 Fig and S1 Table).

## Discussion

The major findings of this study are: 1) the C allele rs9508025 in *FLT1* is significantly associated with a higher MACE rate, 2) the genotype of rs1333049 in 9p21 did not show an association with the composite outcome, 3) the association of the rs9508025 variant with MACE was mainly due to its relation to coronary revascularization, which was also associated with the rs1333049 variant, 4) the association of rs9508025 was significant only in the subgroup with a history of CAD. The strengths of our study are as follows 1) this study is the first to show the association between an *FLT1* variant and the long-term cardiovascular outcome, 2) we verified the influence of the variant identified in a prior GWAS in Asians, a less-studied ethnicity, 3) to the best of our knowledge, this is one of the largest studies with the longest follow-up evaluating the cardiovascular outcome, 4) in contrast with the prior studies, looking into the individual components of MACE has helped us understand the relation between the variants and specific complications. Our results may provide a novel genomic marker for atherosclerotic cardiovascular disease.

In the current study, we demonstrated the association between rs9508025, a *FLT1* variant and cardiovascular risk, not using case-control design but analyzing clinical follow-up data. To date, studies of the variants in *FLT1* have been conducted mostly in non-cardiovascular diseases. Several SNPs were associated with nephropathy [22], macular degeneration [23], obstructive pulmonary disease [24], cancers [24–26], and the response to anti-cancer therapy [27]. Conversely, studies that investigated relationships between the variants of *FLT1* and CAD are extremely limited. An association between rs931428 of *FLT1* and CAD was discovered in the study by CARDIoGRAMplusC4D Consortium [5], in which a large part of the participants were of European descendants. This marker was recently confirmed in Japanese reports [28]. However, those studies did not find an association between rs9508025, the marker in the current study, with CAD. Due to this disparity among different populations, the value of our marker on cardiovascular outcomes may not be directly applicable to other populations. The risk allele shown in the current study was a C allele that was associated with an increased risk of CAD in our prior report [18]. That report showed the risk analyzed by GWAS and replication by using data of CAD patients and controls. Meanwhile, the present study analyzed the long-term cardiovascular risk. Although long-term outcome is a rather larger concept than CAD risk, we can tell that the directionality of the association between the risk allele and the phenotype is the same. Particularly, many events demanding coronary revascularization are related to the progression of CAD in the same or other coronary arteries [29].

The mechanism underlying the association between *FLT1* variant and CAD or MACE is not fully understood yet. It was reported that a relationship exists between soluble Flt-1, coded by that gene, and mortality in chronic kidney disease patients [30], but the data have been insufficient to explain its biological effect. A variant of rs9319428 in *FLT1* revealed an association with diastolic blood pressure [31], however, in our current study, the relationship between rs9508025 variants and blood pressure was not found to be significant (data not shown). A functional analysis of pathways of genes associated with CAD showed that *FLT1* belongs to the clusters of genes coding proteins involved in focal adhesion and extracellular matrix interactions [32]. In atherosclerotic cardiovascular disease, ischemic episodes are critically influenced by collateral circulation rather than atheroma burden only [33]. Therefore, it cannot be ruled out that *FLT1* codes a protein that plays a role in angiogenesis and vascular development [34], which influence cardiovascular risk. Our results showed that a *FLT1* variant (rs9508025) was related to the incidence of coronary revascularization. The occurrence of revascularization depends on many factors, including the progression of arterial stenosis, thrombotic events, and even physician’s discretion. Therefore, it is difficult to explain, using our current data, how this variant was linked to the rate of revascularization.

Ever since it was first reported in 2007, the association between 9p21 and CAD risk has been steadily replicated in various ethnicities [5]. In addition, the significance of several SNPs in 9p21, such as rs1333049, rs2383206, and rs10757278 were validated in East Asians as well [15]. Although our data showed that the C allele of rs1333049 correlates with the baseline CAD risk, the association of this SNP was not significant for the long-term outcomes. Studies of the influence of variants in 9p21 on the clinical outcomes have been limited, and their results were inconsistent. Thus, it seems premature to come to any conclusion about the relationship between this locus and the long-term cardiovascular outcomes. In the studies performed in Germany [35], China [16], New Zealand [36], or USA [37], rs1333049 was not found to be associated with total mortality or cardiovascular events. On the contrary, this SNP was linked to the incidence of cardiovascular diseases in the Bruneck study with a longer follow-up [38]. Similar finding has also been observed in the MORGAM project [12]. In general, however, most of the studies did not follow up the patients sufficiently long, and the cardiovascular events were not analyzed in detail according to the individual components. We analyzed the association between each SNP and the individual components of MACE, and determined that a variant of rs1333049 correlates with the incidence of coronary revascularization. This finding may be in accordance with previous studies that showed associations between 9p21 variants and the CAD burden [39, 40] or the progression of coronary atherosclerosis [41].

This study has several potential limitations in addition to its strengths. We analyzed the effects of two SNPs selected based on a prior GWAS. Evaluation of more loci may have provided us with additional information about their association with cardiovascular events. Furthermore, data on pharmacological or non-pharmacological treatments were not available in this study. Therefore, we cannot fully rule out the possible influences of those therapeutic factors. However, the frequencies of risk factors were similar between the individuals with different genotypes, so the difference in treatments between different groups may have been small. In addition, statistical significance of our findings after correction may not be sufficiently strong. Also, p-values were not corrected for the number of traits tested and should be interpreted with caution.

## Conclusions

In conclusion, rs9508025 in FLT1 was significantly associated with long-term cardiovascular events, particularly in patients with CAD. This relationship was mainly due to its influence on coronary revascularization. The association of rs1333049 in 9p21 with the outcome was not significant, although this variant was also linked to the risk of coronary revascularization.

## Supporting Information

S1 Fig **Kaplan-Meier curves for the long-term MACE-free survival in the subgroup with CAD according to the rs9508025 (A) and rs1333049 polymorphisms (B).** The impact of rs9508025 on MACE was evident in the subgroup with CAD, whereas rs1333049 did not show significant effect.
(TIF)

S1 Table Predictors of the individual components of MACE in patients with or without CAD. (DOCX)
