# Supplementary figures and images for: The Effect of FLT1 Variant on Long-Term Cardiovascular Outcomes: Validation of a Locus Identified in a Previous Genome-Wide Association Study
Source: PLoS One. 2016 Oct 13;11(10):e0164705. doi: 10.1371/journal.pone.0164705 (PMC5063388; doi:10.1371/journal.pone.0164705)

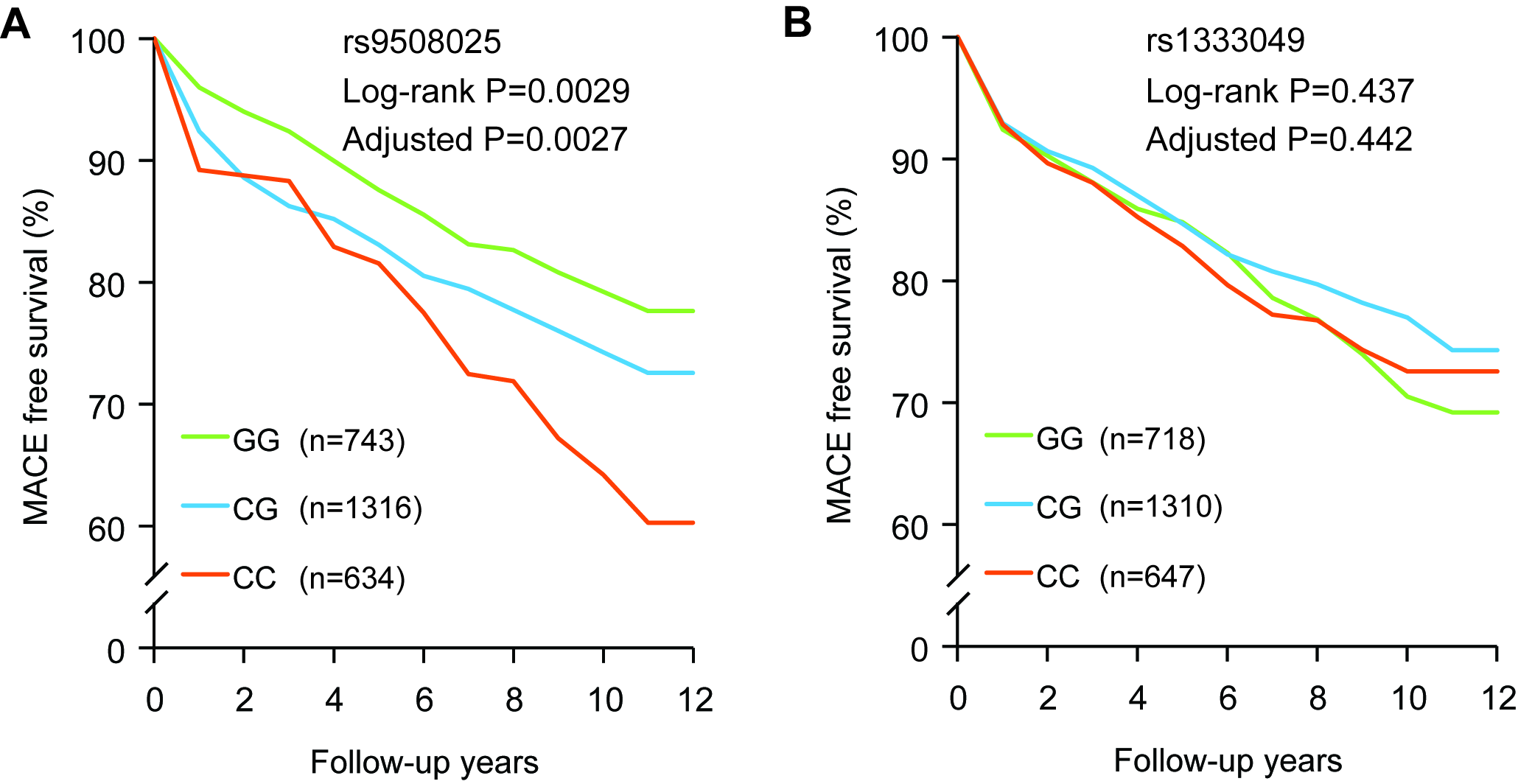

Supplement: S1 Fig — Kaplan-Meier curves for the long-term MACE-free survival in the subgroup with CAD according to the rs9508025 (A) and rs1333049 polymorphisms (B). The impact of rs9508025 on MACE was evident in the subgroup with CAD, whereas rs1333049 did not show significant effect. (TIF) [file pone.0164705.s001.tif]
