# Supplementary material for: The Effect of FLT1 Variant on Long-Term Cardiovascular Outcomes: Validation of a Locus Identified in a Previous Genome-Wide Association Study
Source: PLoS One. 2016 Oct 13;11(10):e0164705. doi: 10.1371/journal.pone.0164705 (PMC5063388; doi:10.1371/journal.pone.0164705)
Supplement: S1 Table — (DOCX) [file pone.0164705.s002.docx]

**Supporting Information**

**S1 Table. Predictors of the individual components of MACE in patients with or without CAD**

|  | Patients with CAD | | Patients without CAD | |
| --- | --- | --- | --- | --- |
| Factor | HR (95% CI) | P | HR (95% CI) | P |
| Age | 1.00 (0.98-1.02) | 0.97 | 1.05 (1.04-1.07) | <.001 |
| Male | 1.55 (1.08-2.21) | 0.02 | 1.24 (0.90-1.69) | 0.19 |
| Hypertension | 1.50 (0.37-6.08) | 0.57 | 1.20 (0.38-3.82) | 0.75 |
| Diabetes mellitus | 1.28 (0.91-1.79) | 0.15 | 1.89 (1.29-2.77) | 0.001 |
| Hyperlipidemia | 1.19 (0.87-1.62) | 0.28 | 1.11 (0.81-1.52) | 0.53 |
| Current smoker | 1.20 (0.83-1.75) | 0.34 | 0.95 (0.59-1.53) | 0.84 |
| rs9508025^a^ | 1.39 (1.12-1.72) | 0.003 | 1.04 (0.85-1.28) | 0.70 |
| rs1333049^a^ | 1.08 (0.88-1.33) | 0.44 | 1.07 (0.86-1.32) | 0.55 |

MACE: major adverse cardiac events; CAD: coronary artery disease; HR: hazard ratio; CI: confidence internal

^a^Additive genetic model
